# Late Hip Displacement Identified in Children at Gross Motor Function Classification System II and III With Asymmetric Diplegia and Fixed Pelvic Obliquity

**DOI:** 10.5435/JAAOSGlobal-D-20-00094

**Published:** 2022-09-21

**Authors:** Stacey Miller, Lise Leveille, Maria Juricic, Kishore Mulpuri

**Affiliations:** From BC Children's Hospital (Ms. Miller and Ms. Juricic); Department of Physical Therapy, University of British Columbia, Vancouver, BC, Canada (Ms. Miller and Ms. Juricic); the Department of Orthopaedic Surgery, BC Children’s Hospital (Dr. Leveille and Dr. Mulpuri); and the Department of Orthopaedics, University of British Columbia (Dr. Leveille and Dr. Mulpuri).

## Abstract

Risk of hip displacement in children with cerebral palsy is directly related to a child's level of motor function as classified by the Gross Motor Function Classification System (GMFCS) and is reported to be greatest at a young age. In this study, we present a series of four children with asymmetric diplegic cerebral palsy at GMFCS levels II and III, with the more involved hip showing rapid, progressive displacement at a later age. Current hip surveillance guidelines may not adequately identify hip displacement in children with asymmetric diplegia and pelvic obliquity; modifications to surveillance guidelines may be warranted. Additional investigation of hip displacement in this subset of children is required to determine whether the incidence of displacement is higher than anticipated based on the GMFCS level alone.

Hip displacement in children with cerebral palsy (CP) is a common problem, with one in three children being affected.^1-3^ Systematic hip surveillance is now considered the standard of care for children with CP to ensure that displacement is detected early. The frequency of surveillance is based on Gross Motor Function Classification System (GMFCS) level, age, and, for children with hemiplegia, their gait pattern.

The correlation between risk of hip displacement and GMFCS level is well established.^1-3^ Children whose motor function is classified at level I have very low risk of hip displacement while those classified at level V have up to 90% risk. Risk of displacement is greatest at the age of 3 to 5 years.^4-6^ However, late progression may also occur and justifies continuing surveillance until skeletal maturity for children at higher risk GMFCS levels.^7,8^ In children with hemiplegia, a group IV gait pattern studied by Winters et al has been associated with increased risk of progressive displacement at a later age.^9,10^ This gait pattern is characterized by a flexed hip and anteriorly tilted pelvis in the sagittal plane and an adducted and internally rotated hip in the coronal and transverse planes.^10,11^ Although the incidence of hip displacement in children with this gait pattern is unknown, separate recommendations for surveillance are available.^9,12,13^ Based on the GMFCS level, most children with a group IV hemiplegic gait pattern would be discharged from surveillance by the age of 10 years, but, when this pattern is identified, current guidelines in Australia and North America recommend surveillance continue with clinical examinations and radiographs obtained every 12 or 24 months until skeletal maturity.^9,12,13^

Gait patterns in children with diplegia have not been described as a potential risk factor of hip displacement. Rodda et al^14^ defined five sagittal gait patterns in children with diplegic CP. One of these, an asymmetric pattern, is described to occur when each lower limb displays a different gait pattern in the sagittal plane (true equinus, jump, apparent equinus, or crouch). Differences in the transverse and coronal planes are not considered in this definition. However, all three planes of motion are evaluated during instrumented gait analysis and are important when assessing asymmetry.^15^ We question whether children with diplegia displaying an asymmetric gait pattern may be at increased risk of progressive displacement on their more affected side.

This case series describes four ambulant children with asymmetric diplegia who were identified through a tertiary care pediatric orthopaedic clinic as having late progressive hip displacement. All had undergone prior video or instrumented gait analysis and had serial AP pelvic radiographs. Pelvic imaging was reviewed to determine the age at which hip displacement, defined as Reimer migration percentage (MP) >30%, was identified and the rate of progressive displacement. Gait laboratory graphs of the kinematic data for the hips and pelvis of all four children were visually reviewed to look for similarities across children. Current hip surveillance guidelines may fail to identify the late, rapid hip displacement occurring in children with asymmetric diplegia as demonstrated here.

## Case Series

**Figure 1:**
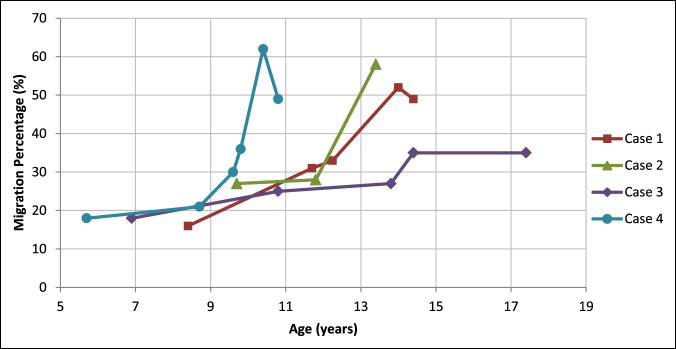
Graph showing change in migration percentage (MP) of the most involved hip for the four case series children. Note: The drop in MP for case 4 was due to poor positioning of the fifth image (62%), with the hip significantly adducted.

**Figure 2:**
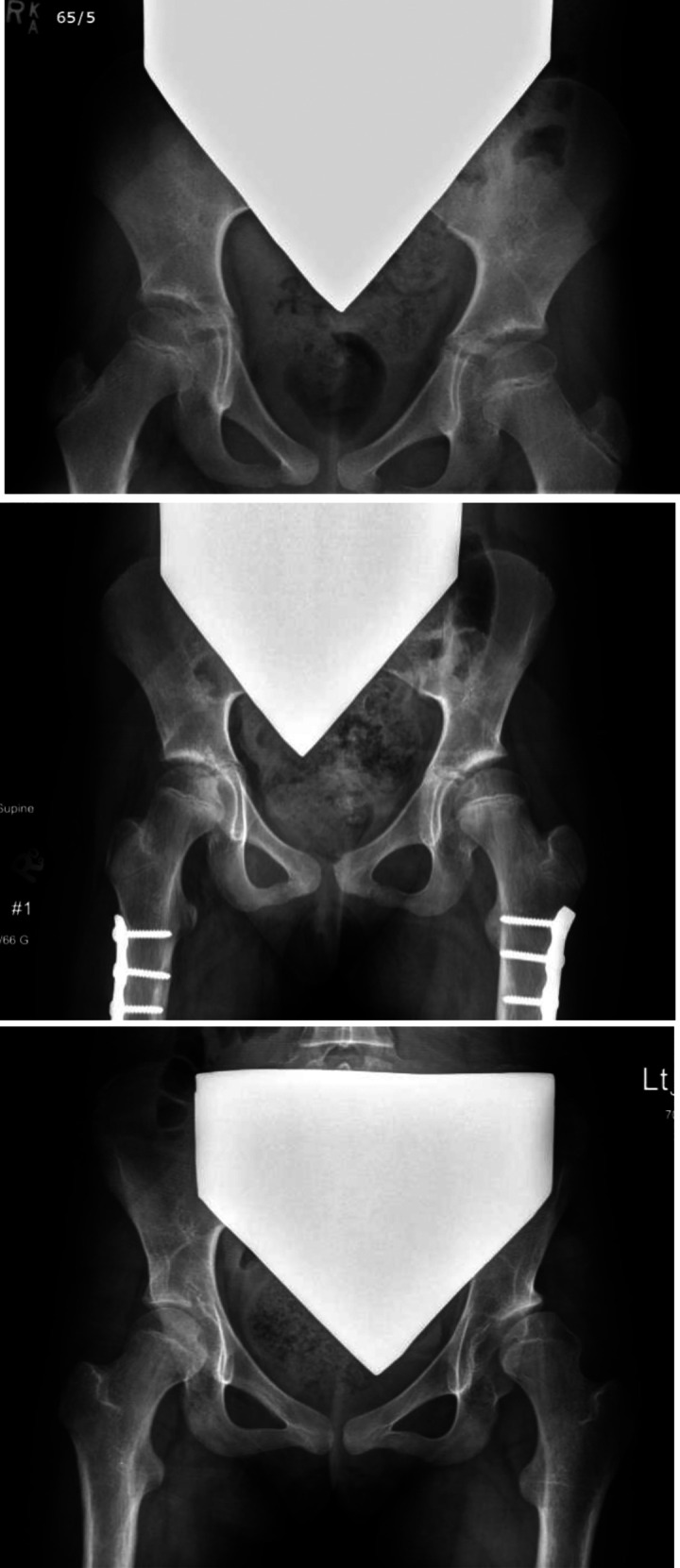
Radiographs of youth in case 1 aged 8.4 years, 11.7 years, and 14.0 years showing late progression of hip displacement of the left hip and erosion of the femoral head.

**Figure 3:**
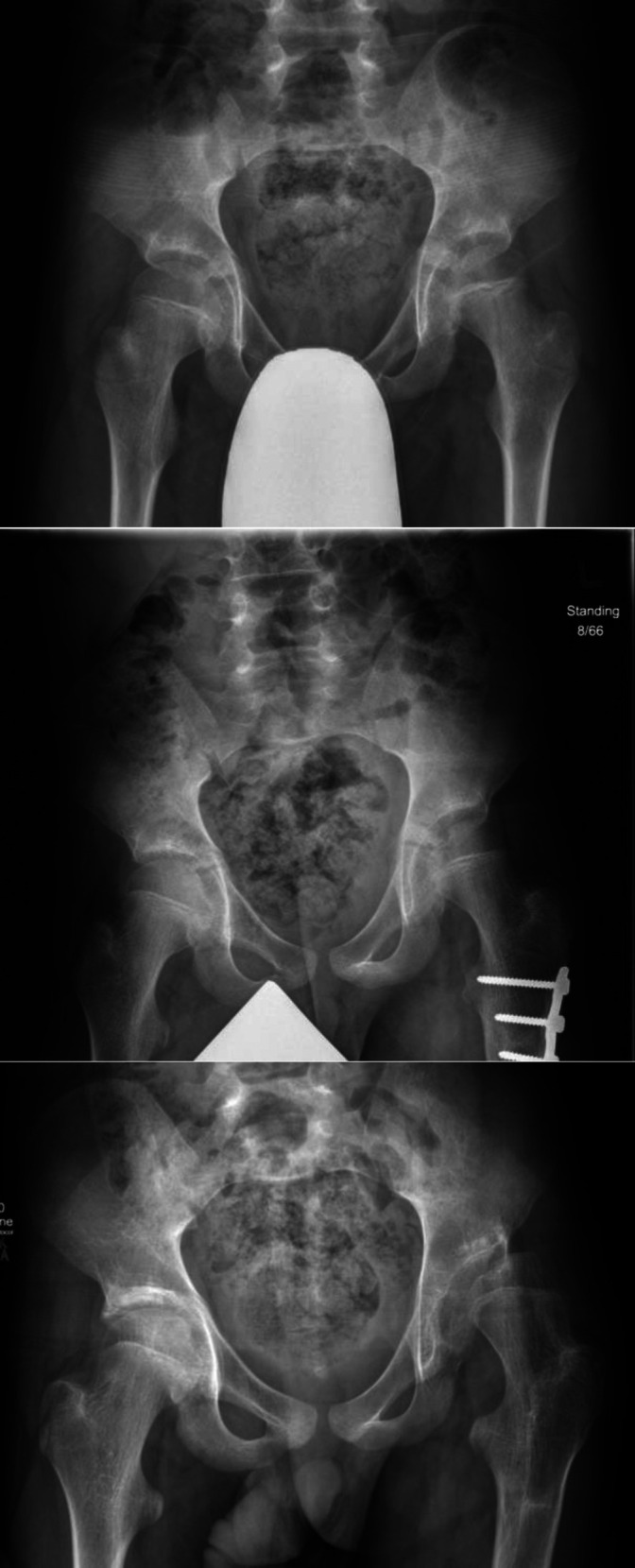
Radiographs of youth in case 2 aged 9.7 years, 11.6 years, and 13.4 years showing late progression of hip displacement of the left hip and erosion of the femoral head.

Clinical and radiographic characteristics of each case are listed in Table 1. Continuous variables were described with means, where appropriate. The more involved side was identified based on clinical examination, including assessment of range of motion, muscle tone, and strength. Change in MP of the more affected hip, measured from serial AP pelvic radiographs, is shown in Figure 1. In all children, the MP of the less involved hip has remained less than 30%, ranging from 10% to 15% (mean 11%) at the last follow-up. The first radiographic evidence of hip displacement, defined as a MP of >30%, occurred at a mean age of 12.3 years (range 9.5 to 14.4 years). At the most recent follow-up or before surgical intervention for hip displacement, mean MP measured 48% (range 35% to 58%). All demonstrated rapid progression in MP with increases of 19%, 30%, 13%, and 8% in a period of 20, 18, 11, and 7 months, respectively (Figure 1). In Figures 2 and 3, AP pelvic radiographs of case 1 and case 2 illustrate their progressive hip displacement. Erosion of the lateral femoral head was noted in these two children. Three of the four children reached the surgical threshold for reconstructive surgery, with surgery planned or completed. In three cases, pelvis obliquity on supine AP pelvic radiographs was evident, and increasing scoliosis was noted in two of these (Table 1). Leg-length discrepancy was less than 2 cm in all patients.

**Table 1:** Demographic and Radiological Data for Each children

Factor	Case 1	Case 2	Case 3	Case 4
Sex	Female	Male	Male	Male
GMFCS level	II	III	II	III
More involved side	Left	Left	Right	Right
Age, MP, and AI/AA values (right, left) when first MP >30%	11.7 yr 8%, 31% 8°, 15° (AI)	13.4 yr 10%, 58% 43°, 49° (AA)	14.4 yr 35%, 16% 54°, 37°, (AA)	9.6 yr 30%, 0% 14°, 10° (AI)
Age, MP, and AI/AA values (right, left) at the last radiograph^a^	14.4 yr 15%, 49% 43°, 47° (AA)	13.4 yr 10%, 58% 43°, 49° (AA)	17.4 yr 35%, 10% 52°, 38° (AA)	10.8 yr 49%, 10% 19°, 7° (AI)
Femoral head erosion	Yes	Yes	No	No
Pain	No	Yes	No	Yes
Pelvic obliquity on AP pelvis radiograph	11.7 yr: 0° 14.4 yr: 0°	13.4 yr: 6° (left high)	14.4 yr: 11° (right high) 17.4 yr: 9° (right high)	9.6 yr: 8° (right high) 10.8 yr: 4° (right high)
Age and Cobb angle on scoliosis imaging (standing)	11.7 yr: 10° thoracic	13.0 yr: 14° thoracic, 10° lumbar 15.8 yr: 30° lower thoracic/lumbar	14.4 yr: 10° thoracic 17.4 yr: 5° thoracic	10.5 yr: 19° thoracolumbar (sitting)
Reconstructive hip surgery	Planned	Complete (13.4 yr)	No	Complete (10.8 yr)

AA = acetabular angle (closed triradiate cartilage), AI = acetabular index (open triradiate cartilage), GMFCS = Gross Motor Function Classification System, MP = migration percentage

a MP at the last preoperative radiograph for patients who underwent reconstructive hip surgery.

**Figure 4:**
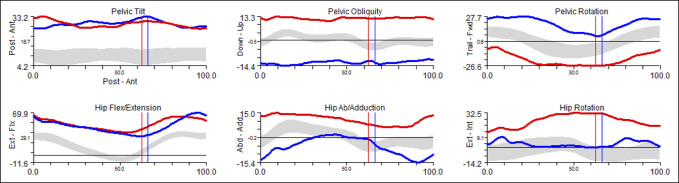
Examples of kinematic graphs of the pelvis and hip.

Observations from instrumented gait laboratory data are summarized in Table 2. Kinematic data showed that all children had pelvic obliquity with the more involved side high. The obliquity was fixed in all children. In case 1, the fixed pelvic obliquity developed; initially, the child had a Trendelenburg gait and fell into the oblique pelvic position after loading response. The more involved hemipelvis trailed. Three had notable internal rotation on the more involved side. The remaining children had an internal foot progression angle on the more involved side because of the position of the pelvis. Before the onset of progressive hip displacement, three of the children underwent subtrochanteric femoral derotation surgery, without varus, between the age of 8.8 and 10.3 years to correct excessive femoral internal rotation. In the two children with notable preoperative internal rotation of the more involved hip (cases 1 and 3), the increased internal rotation reoccurred. Increased hip flexion in stance was seen in the kinematic data of two children but was not severely asymmetric between the more and less involved sides. An example of kinematic graphs of the pelvis and hip are shown in Figure 4.

**Table 2:** Gait Laboratory Data for Each children

Case No.	More Involved Side	Age (yr)	Gait laboratory findings		
			Pelvic Obliquity (PO)	Trailing Hemipelvis	Average Internal Hip Rotation in Stance (Right, Left)
1	Left	8.9	Bilateral Trendelenburg; no PO	Left	17°, 25°
		12.8	Fixed; left high	Left	3°, 42°
		9.5 × gait surgery − bilateral femoral derotation osteotomies + botulinum toxin			
2	Left	10.1	Fixed; left high	Right	19°, 7°
		11.8	Fixed; left high	Right	3°, 0°
		10.3 × gait surgery − left femoral derotation osteotomy + right hamstring release, bilateral rectus femoris releases, bilateral calcaneal lengthenings			
3	Right	8.4	Fixed; right high	Right	17°, 3°
		12.0	Fixed; right high	Right	5°, 6°
		15.7	Fixed; right high	Right	31°, −5°
		8.8 × gait surgery − right femoral derotation osteotomy + left calcaneal lengthening			
4	Right	9.5	Fixed; right high	Right	23°, 4°

## Discussion

This case series is the first to report rapid, progressive hip displacement in ambulatory patients with asymmetric diplegic CP. Although the rate of increase in MP in children with CP by the GMFCS level is not clearly defined in the literature, Pruszczynski et al^5^ reported that the percentage increase in MP for children at GMFCS levels II and III is less than 5% per year. All hips in this case series had substantially higher rates of progression (Figure 1) than this reported value. In addition, hip displacement in this series occurred later than the documented highest risk ages. In all cases, hip displacement occurred at an age at which standard radiographic surveillance would not have been indicated based on American, Canadian, and European surveillance recommendations.^5,12,13,17^ Possibly, more frequent and prolonged radiographic follow-up may be useful for patients with pelvic obliquity and asymmetric diplegia.

On instrumented gait analysis, all children demonstrated a fixed pelvic obliquity, with the more involved side high. The hemipelvis on the more involved side usually trailed. Most had increased hip internal rotation on the more involved side. Even when corrected with femoral derotation osteotomies, the excessive internal rotation on the more involved side had recurred at the last follow-up. These gait findings are consistent with those of a Group IV hemiplegic gait pattern.

On radiographic analysis, pelvic obliquity, measuring between 4° and 11°, was evident on supine AP pelvic imaging in all but one subject. The more involved side was high, consistent with the gait laboratory findings. In a review of pelvic obliquity in adolescents and young adults with CP, Heidt et al found that as pelvic obliquity increased, the migration percentage increased in the hip on the high side and decreased on the low side. They reported a similar case of a young adult at GMFCS level III with unilateral hip displacement and pelvic obliquity on pelvis imaging and gait analysis.^16^

Two children, both at GMFCS level III, developed notable curves involving the lumbar spine. In one of these children (case 2), the lumbar curve was present at the time the MP exceeded 30%, measuring 10°. Possibly, scoliosis was a contributing factor leading to pelvic obliquity and subsequent hip displacement. In the two remaining cases, scoliosis was not present as a potential cause of pelvic obliquity. The presence of a scoliosis in these two children suggests that they may have more severe musculoskeletal involvement, and it is possible that this, rather than their gait pattern, is contributing to the observed progressive hip displacement.

Based on existing hip surveillance guidelines, children at GMFCS level II may be discharged from surveillance between the age of 6 and 10 years.^9,12,13,17^ Some surveillance guidelines provide separate recommendations for children with a Group IV hemiplegic gait pattern, recommending that children with this gait pattern receive monitoring until skeletal maturity. Similar guidelines are not available for children with asymmetric diplegia, and there is risk that children in this case series may have been missed based on current surveillance guidelines. Increased frequency or prolonged surveillance may be indicated for children with asymmetric diplegia with a type IV hemiplegic pattern on the more affected side. Although recommendations suggest children at GMFCS level III be followed to skeletal maturity, reducing surveillance for this subgroup to every 2 years may result in a delay in the identification of rapid progression, as seen here. When reviewing the Australian guidelines, Wynter et al^9^ recommended that children be followed annually in the presence of pelvic obliquity, leg-length discrepancy, or deteriorating gait. These are prudent recommendations and may have enabled the hip displacement described in these cases to be detected earlier.

This case series suggests that youth with asymmetric diplegia who demonstrate a fixed pelvic obliquity through stance, with the more involved side high, may be at increased risk of hip displacement. An internally rotated hip and trailing hemipelvis may also be of significance. However, this case series includes only four patients, and a consecutive series of children with asymmetric diplegia has not been reviewed. We are now conducting a review of all children at GMFCS levels II and III with asymmetric diplegia who demonstrate pelvic and hip asymmetries to see whether their rates of hip displacement differ from what have been reported based on the GMFCS level alone. Similarly, additional characterization of gait kinematics that may be used to aid in identifying children at increased risk in a larger cohort of children is being explored. This work will aid in determining whether the relative risk of hip displacement in children with diplegia differs based on gait pattern and pelvic obliquity and whether changes in hip surveillance guidelines are warranted.
